# Experiences of dental care: what do patients value?

**DOI:** 10.1186/1472-6963-12-177

**Published:** 2012-06-24

**Authors:** Alexandra Sbaraini, Stacy M Carter, R Wendell Evans, Anthony Blinkhorn

**Affiliations:** 1 Centre for Values, Ethics and the Law in Medicine, University of Sydney, Sydney, NSW, Australia; 2 Population Oral Health, Faculty of Dentistry, University of Sydney, Sydney, NSW, Australia

**Keywords:** Qualitative research, Dentist-patient relationship, Prevention

## Abstract

**Background:**

Dentistry in Australia combines business and health care service, that is, the majority of patients pay money for tangible dental procedures such as fluoride applications, dental radiographs, dental fillings, crowns, and dentures among others. There is evidence that patients question dentists’ behaviours and attitudes during a dental visit when those highly technical procedures are performed. However, little is known about how patients’ experience dental care as a whole. This paper illustrates the findings from a qualitative study recently undertaken in general dental practice in Australia. It focuses on patients’ experiences of dental care, particularly on the relationship between patients and dentists during the provision of preventive care and advice in general dental practices.

**Methods:**

Seventeen patients were interviewed. Data analysis consisted of transcript coding, detailed memo writing, and data interpretation.

**Results:**

Patients described their experiences when visiting dental practices with and without a structured preventive approach in place, together with the historical, biological, financial, psychosocial and habitual dimensions of their experience. Potential barriers that could hinder preventive activities as well as facilitators for prevention were also described. The offer of preventive dental care and advice was an amazing revelation for this group of patients as they realized that dentists could practice dentistry without having to “drill and fill” their teeth. All patients, regardless of the practice they came from or their level of clinical risk of developing dental caries, valued having a caring dentist who respected them and listened to their concerns without “blaming” them for their oral health status. These patients complied with and supported the preventive care options because they were being “treated as a person not as a patient” by their dentists. Patients valued dentists who made them aware of existing preventive options, educated them about how to maintain a healthy mouth and teeth, and supported and reassured them frequently during visits.

**Conclusions:**

Patients valued having a supportive and caring dentist and a dedicated dental team. The experience of having a dedicated, supportive and caring dentist helped patients to take control of their own oral health. These dentists and dental teams produced profound changes in not just the oral health care routines of patients, but in the way patients thought about their own oral health and the role of dental professionals.

## Background

This study was built on a previous randomized controlled trial (RCT) undertaken in private general dental practices in New South Wales (NSW), Australia [1]. During the previous RCT, 22 practices were randomly allocated to either the intervention (n=12) or the control group (n=10). A total of 847 patients were recruited (intervention group n = 427; control group n = 420) within the 22 dental practices between May 2005 and March 2006 [1].

Intervention practices in the RCT were provided with evidence-based preventive protocols to offer a less invasive approach to the treatment of dental caries [2]. The protocols advised dentists to systematically apply preventive techniques to prevent new dental caries and to arrest the early stages of dental caries, thereby reducing the need for restorative care. The protocols focused on primary prevention of new dental caries (via tooth brushing with high concentration fluoride toothpaste and dietary advice) and intensive secondary prevention through professional treatment to arrest dental caries progress (applying fluoride varnish and monitoring the success of tooth brushing by recording the levels of dental plaque on the teeth)[2].

During the RCT, the numbers of decayed, missing and filled teeth (DMFT) were monitored over time. The RCT final results showed a highly significant difference in the incremental DMFT score in favour of the intervention group (two-year mean difference: 0.8; p < 0.001, three-year mean difference: 0.9; p < 0.001) [3]. Patients in the intervention group had fewer repeat dental fillings at both two (p < 0.001) and three (p < 0.001) years [3]. Having an increased risk of developing dental caries was observed in 11% of patients in the intervention group compared to 24% in the control group (p < 0.001) [3]. Dentists, members of dental team and patients from the practices involved in the RCT were asked to participate in this qualitative study [4].

### The context of this study: Dentists and patients operating in a typical Australian clinical context

Dentistry as practiced in Australia combines business and health care. More than 80% of dentists work in private general dental practices [5]. General dentists provide the majority of care and dental hygienists are employed in only a minority of practices [5,6]. The majority of dentists are independent self-employed practitioners; they own their practices and lead their dental team.

Apart from its private practice essence, dental services differ from other outpatient health care fields because of a focus on providing tangible treatments; patients leave a dental practice fully aware that procedures were done in their mouths, and sometimes are told to re-attend for further physical interventions in follow-up appointments. This is in contrast to a visit to a doctor where the focus might be receiving health advice, routine exams and/or drug prescriptions.

In Australia, most people pay for their own dental treatments, or for the private health insurance that partly covers the cost of dental care [7]. The majority of adults in NSW visit a private general dental practice for a check-up at least once a year on average; residents outside capital cities visit less frequently [7]. Most individuals visit the same private dental practitioner on a long term basis [8].

This study focused on dentists and patients in private general dental practices – that is, on dentists and patients operating in a typical Australian clinical context.

### Measuring patients’ satisfaction and expectations of care

‘Patient satisfaction’ is generally conceptualized as a construct that can be measured with standardized quantitative instruments and compared between sites or treatments. These instruments are often modified for use in specific settings or topic areas, with primary research and systematic reviews conducted regarding patient satisfaction with particular types of treatment. For example, in dentistry, questionnaire surveys have been used to evaluate patients’ uneasiness before treatment, their dislikes during treatment as well as their opinions about certain aspects of the service such as dentists’ technical ability, treatment costs and service facilities [9,10]. In the medical literature, there is also some research into the relationship between satisfaction and general aspects of care that are shared across different clinical contexts such as shared decision making [11]. In addition, a parallel stream of qualitative and social research provides a broad-based outlook while seeking to understand patients’ experiences of care on patients’ own terms. Entwistle and colleagues recently completed an interpretive synthesis of this literature and argued that “the characteristics and actions of health care services and staff, and the ways they relate to patients, have implications for patients’ experiences of being enabled (or not) to feel, be and do what they value feeling, being and doing – in the course of their health care contacts and beyond. Experiences of health care delivery matter because they shape and represent capabilities that are key to how well people’s lives can go” [12], p4]. Therefore, this study is focused on understanding dental patients’ experience on its own terms, rather than evaluating patient satisfaction using standardized methods.

### What do we know about patients’ expectations when visiting a dental practice?

The dentist-patient relationship literature provides some clear advice about patients’ expectations and perceptions when visiting a dental practice. These expectations are less related to the technical competence of dentists, and more to do with the attitudes and communication skills [9,13-16]. In particular patients want a dentist who listens to them, has a friendly caring attitude, explains treatment options and procedures, and inspires confidence [9,15,17]. This is consistent with research findings in the medical literature which shows that “the most important health service factor affecting” patient satisfaction is the quality of the doctor-patient relationship [18].

While we know from the dental literature what patients expect from their dentists, it is not clear how patients experience different approaches to treat dental caries, what hurdles they might encounter when asked to change their routines to comply with self-care recommendations, and what is important and valued during the dental care experience. This paper reports on one aspect of the overall qualitative study: patients’ experiences of dental care, particularly on the relationship between patients and general dental practitioners during the provision of preventive care and advice, and on what patients valued in dental care. Accordingly, the final research questions for this component of the study were:

1) What was patients’ experience of dental care in practices without a structured approach to prevention?

2) What was patients’ experience of dental care in practices with a structured approach to prevention?

3) What were the barriers and facilitators for prevention for these patients?

4) What did these patients value in dental care?

## Methods

### Study design

A previous paper has described the sampling, data collection, analysis and interpretation in detail [4]. During the study, Charmaz’s grounded theory methodology was employed to examine the social process of adopting preventive dental care in dental practices [19]. Charmaz’s methodology suggests a systematic set of procedures to study and understand social processes, actions and interactions between individuals [19]. Accordingly, we sought to learn from patients how the process of adopting prevention worked and how they made sense of it. Throughout the study it was important to acknowledge that as researchers we had some pre-existing concepts in mind due to our academic backgrounds in dentistry and public health, although we deliberately remained open to what patients would tell us about their experiences [4].

### Sampling strategy

Two dental practices (Dental Practice 1 and 2) which had offered the preventive care program consented to send letters of invitation to participate in this study to all patients previously enrolled in the RCT. Patients who agreed to participate in the study differed based on their clinically measured risk of developing dental caries: some patients whose risk status had decreased, some whose risk had increased and some whose risk had stayed the same over the previous RCT study were interviewed (Table 1). This purposive sampling allowed comparisons between dental care experiences of patients with different clinical outcomes, as we expected that this might be different. After analysing the first round of interview data from Dental Practice 1, patients from Dental Practice 2 were interviewed. This allowed comparisons between patients in a practice where the preventive protocols were successfully implemented (Dental Practice 1), and those who were treated in a practice where the program had been less successful (Dental Practice 2).

**Table 1:** Patients’ characteristics (n = 17)

**Patients characteristics**	**Dental Practice 1**	**Dental Practice 2**
Number of patients interviewed	N = 12	N = 5
Option of location for interview	At dental practice: n = 4 At community centre: n = 4 At patients’ home: n = 1 By phone: n=3	By phone: n = 5
Age range	18-65 years old	25-55 years old
Gender	Female: n = 7 Male: n = 5	Female: n = 3 Male: n = 2
Risk of developing dental caries	Decreased: n = 6 Increased: n = 3 Stayed the same: n = 3	Decreased: n = 1 Increased: n = 2 Stayed the same: n = 2

### Sample size and saturation

Qualitative researchers generally seek to reach ‘saturation’ in their studies [4]. Often this is interpreted as meaning that the researchers are hearing nothing new from patients during interviews. In a grounded theory study, theoretical saturation is sought [4]. This is a subtly different form of saturation, in which all of the concepts in the substantive theory being developed are well understood and can be substantiated from the data [19]. Accordingly, saturation is determined by the data analyst. When new interviews become repetitive with prior interviews and central concepts are fully understood, the analyst determines that saturation was reached [20]. In our study, data from the last four patients interviewed (two from each dental practice sampled) confirmed our findings rather than adding new concepts. Therefore data collection ceased. In total 17 patients, ranging in age from 18 to 65 years old, participated in the interview process (Table 1).

### Interviews

Patients were interviewed for approximately one hour in locations convenient to them such as dental practices or homes. Some preferred to be interviewed over the phone, when the same format was used as for face to face interviews. Sturges and Hanrahan have reported that telephone interviews give the same in-depth data as face to face interviews [21]. The semi-structured interviews were digitally recorded, professionally transcribed in detail, and the transcripts were checked against the recordings. Table 2 details questions that guided interviews. The researcher/interviewer (AS) explored how patients experienced dental care, what dental care in general and preventive care meant to patients, how and why they did or did not adopt the prescribed preventive care, and how this was influenced by their social context. Interviews were conducted between October 2009 and November 2010.

**Table 2:** Examples of questions asked during interviews

**Opening questions**	**We are going to start by talking about oral health.**
	When I say oral/dental health, what is the first thing that comes to your mind?
	Everyone’s experience of oral/dental health is different. In your own case:
	▪How would you describe your oral/dental health at the moment?
	▪How important to you is attending a dental appointment? Why?
	▪In general, what do you expect to get from your dental appointment?
	▪Could you describe a typical visit to the dentist?
	▪What is it that you like about seeing the dentist?
	▪What is it that you dislike about seeing the dentist?
**Transitional questions**	**Now we are going to talk about changes to your oral health and dental care.**
	If you think back in the last year, how many times did you visit the dentist?
	Could you tell me about what led you to go or not go?
	Could you tell me what happened during those visits (what kind of treatment?)
	▪Has the care you received changed in the last 2 years?
	▪How has it changed?
	▪What was it like before?
	▪What do you think made it change?
	▪Who and what was important in this process?
	▪How do you feel about this change?
	If you could change the dental care that you receive, how would you change it? How would you like it to be different?
	Over the past two years, your dentist introduced a new system for the treatment of tooth decay.
	▪Do you remember hearing or being told about this system in the practice?
	▪Can you tell me the story of how you found out about it?
	▪How did you feel about it?
	▪Did you have any opportunity to follow the system?
	·If so: what tasks were you able to perform?
	·If so: what made it possible for you to perform those tasks?
	·If so: did it make any difference for you?
**Concluding questions**	**Now I am just going to sum up what I think I have learned about your oral health over the last two years** [SUM UP HERE]. Does that sound right? Now, can I just double check with you to make sure I haven’t missed anything?
	▪Is there anything else that has changed in your relationship with your dentist?
	▪Is there anything else that has changed in your understanding of your teeth?
	▪Is there anything else that has changed in the way you look after your teeth?
	▪Is there anything else you think I should know?
	▪Is there anything you would like to ask me?

### Ethics approval and ethical issues

Initial ethics approval was obtained from the Human Research Ethics Committee at the University of Sydney. As in any ethical study, we ensured that participation was voluntary, that patients could withdraw at any time, and that confidentiality was protected. All responses were anonymised before analysis, and we took particular care not to reveal potentially identifying details of places, practices or clinicians. Prior to being interviewed, all patients had the study explained to them and signed a consent form. It was also explained to patients that their decision to participate (or not) in the study would not affect their relationship with their dentists and dental team.

### Data analysis

#### Coding and the constant comparative method

Charmaz's iteration [19] of the constant comparative method was used during the data analysis. This involved coding of interview transcripts, detailed memo writing and drawing diagrams [4]. The transcripts were analyzed as soon as possible after each round of interviews in each dental practice. Coding was conducted primarily by AS, supported by team meetings and discussions when researchers compared their interpretations.

Coding occurred in stages. In initial coding, we generated as many ideas as possible inductively from early data. In Charmaz’s form of grounded theory, codes take the form of gerunds (verbs ending in ‘ing’) which emphasises actions and processes [19]. In focused coding, we pursued a selected set of central codes throughout the entire dataset and the study [4]. This required decisions about which initial codes were most prevalent or important, and which contributed most to the analysis [4]. In theoretical coding, we refined the final categories and related them to one another [19].

### Memo-writing

The primary analyst also wrote extensive memos which documented the development of the codes, what they meant, how they varied, and how they related to the raw data (transcripts) [4]. Two types of memos were written: case-based and conceptual memos [19]. Case-based memos were written after each interview – containing the interviewer’s impressions about the patients’ experiences and the interviewer’s reactions – memos were also used systematically to question some of our pre-existing ideas in relation to what had been said in the interview [4]. Conceptual memos, on the other hand, were a form of (1) making sense of initial codes; (2) examining patients’ meanings; (3) understanding processes, including when they occurred and changed and what their consequences were. In these memos, we compared data in order to find similarities and differences. Ideas were systematically indexed in memos. This process raised new questions, which were investigated in continuing interviews [4].

## Results

At the beginning of the study, we wrongly assumed that the instructions provided within the RCT would either be implemented or not implemented by patients, and our task would be to understand why they were or were not implemented. Through data analysis however, we realised that what patients were describing was not simply treatment compliance. Patients were talking about a series of issues: their experience when visiting dental practices with and without a structured preventive approach in place; potential barriers that could hinder preventive activities as well as facilitators for prevention, and the nature of the relationship between dentists and patients.

Although we had selected patients with different clinical outcomes, during the course of the study we realized that they were describing similar experiences and sharing the same values about dental care. So, while from the RCT outcomes data it might be reasonable to presume that these patients were not implementing the suggested preventive self-care to the same extent, they still understood dental clinical care in similar ways. During interviews, patients described at length their experience of dental care in practices which they had previously attended. These were dental practices that had not been included in the previous RCT study. Patients compared those experiences with their experiences in the practice they currently attended (Dental Practice 1 or 2), where they had been offered a structured approach to prevention.

We noted in the Methods section that we recruited patients with high, medium and low caries risk, and patients attending two quite different practices. However we note that by the end of the study we were unable to discover any systematic differences between patients, despite careful comparison between them.

### Patients’ experience of dental care in a practice without a structured approach to prevention

During the course of interviews patients wanted to report their earlier experiences of receiving dental care in practices which did not have a structured approach to prevention. It is important to note that those dental practices were not part of the previous RCT study. When spontaneously recalling these past periods, they talked about being trapped in a situation of having “degenerating teeth” and this had historical, biological, financial, psychosocial and habitual dimensions (Table 3).

**Table 3:** Patients’ experience of dental care in practices without a structured approach to prevention

**Being trapped in a situation of having degenerating teeth**		
**Historical dimension:** refers to patients’ dental history, their dental caries experience and fluoride exposure overtime.	Growing up without Fluoride	*“I had gone in and I had a lot of holes because I grew up on a farm with no fluoride.”*
	Having a family history of not having good teeth	*“My mother did not have good teeth and I do not have good teeth. My father has no teeth. He has these bloody ugly, awful bloody false teeth that do not fit him properly. He has had teeth problems all his life, so have I.”*
**Biological dimension:** refers to patients’ experience of dental caries’ clinical signs and symptoms	Having toothache and bleeding gums	*“I had pain and bleeding and when I flossed I used to bleed a lot.”*
	Being someone with poor teeth and losing teeth	*“In the past, I got cavities and then got major problems and lost teeth.”*
**Financial dimension:** refers to the financial burden of dental caries	Forking money out	*"I have been forking money out; because when you are in pain you will pay anything to get the pain to go away.”*
	Not being able to afford restorative treatment	*“I could not afford to go and have my teeth fixed.”*
**Psychosocial dimension:** refers to the psychological and social aspects of patients’ oral health, including patients’ emotional suffering due to dental caries	Wanting to keep my teeth	“*I would like to keep my own teeth and not have false teeth.”*
	Being frustrated	*“It is just disappointing that at certain times I just keep cracking the teeth…”*
**Habitual dimension:** refers to customary activities related to or consequences of dental caries	Being accustomed to have repeated fillings	*“You keep getting more and more fillings in the one tooth.”*
	Being ‘lazy’(oral hygiene)	*“My worst habit is probably not cleaning my teeth regularly before I go to bed, well; I reckon I am a bit lazy.”*

Various aspects of patients’ histories were relevant: family history, personal history, and history of fluoridation. “Having degenerating teeth” – that is, having “poor teeth”, “toothache” and “bleeding gums” – was explained by these historical elements. Patients, who had grown up without fluoride, reported a “family history of bad teeth”, or regretted losing teeth when they were younger. “Having degenerating teeth” had serious implications both in the past and present. Patients also described themselves as “forking money out” due to toothache, or not being able to afford restorative treatment despite being in pain. They wanted to “keep” their teeth, and they were frustrated that their “teeth kept cracking”, but were not necessarily able or motivated to solve the problem. They described themselves as having become accustomed to receiving repeated fillings and being “lazy” about their oral hygiene.

### Patients’ experience of dental care in a practice with a structured approach to prevention

While visiting the dental practices that participated in the previous RCT, patients reported that they no longer felt that they were trapped in a situation of having degenerating teeth as they were able to achieve lifestyle change by working with the dental team. The dimensions shown in Table 3, which had a “degenerative” effect, were being changed into reinforcing outcomes by the preventive program experience (Table 4). Patients realized that preventive care was better than the “old drill and fill” and that they could have solid strong teeth and their general oral health would be better off in the long term. They also understood that preventive care required ongoing changes in their daily routine, took time, and had a cost. However they were “prepared to pay” to “keep their teeth”. More importantly, for the first time these patients felt in personal control of their own oral health and were prepared to brush effectively, use floss and keep regular appointments with the dental team.

**Table 4:** Patients’ experience of dental care in practices with a structured approach to prevention

**Achieving lifestyle change and experiencing reinforcing outcomes**		
**Historical outcomes:** refers to how patients’ dental history changed overtime after being exposed to intensive preventive care.	Having a way to address my dental history	*“Before, I used to go to the dentist if I was in pain or had a broken tooth. Now, I understand that it is not good for me coming to the dentist if my teeth are all falling out – it is a bit late then, right?"*
	Having strong teeth (despite having a family history of poor teeth)	*“I feel more confident now, and my teeth just sort of feel a bit stronger.”*
**Biological outcomes:** refers to patients’ experience of not having dental caries’ clinical signs and symptoms.	Prevention being better than the ‘old drill and fill’	*"It [prevention] is better than going back and having three, or four, or five filling type situations and then going from there.”*
	Having a better outlook	*“Well, to floss, to use the mouthwash, which – yeah, that is good – I like that because it makes you feel cleaner. If you feel clean and comfortable you operate better – your whole outlook is better.”*
**Financial outcomes:** refers to the cost of preventive care and the absence of a financial burden in the long term.	Knowing that it is an ongoing investment	*“I realized that taking care of my teeth is an ongoing thing, but I am prepared to pay for it, if it means keeping my teeth.”*
	Being better off in the long term	*“I am hoping it [prevention] will help me in the longer term with my teeth . Then, I will not need to keep paying for broken teeth to be fixed.”*
**Psychosocial outcomes:** refers to the psychological and social aspects of patients’ improved oral health	Feeling in control	*“I guess emotionally you feel you have addressed that and I am in control now; and I manage it with my regular appointments, the brushing and the flossing.”*
	Feeling satisfied	*“I feel like I have really achieved something, and that is continuing because I am still maintaining and looking after my teeth.”*
**Habitual outcomes:** refers to customary activities related to and/or consequences of preventive care.	Changing visits to dentist	*“Rather than just making an appointment when I got a sore tooth, I was preventing that happening by keeping my regular appointments and having fluoride.”*
	Being part of life	*“I have found that flossing has made quite a big difference, and so I just do that all the time now. It was difficult to start with, but then it was fine; and now it is sort of just a part of life”*

### Barriers and facilitators

While patients valued these reinforcing changes, they also described potential barriers that could have hindered preventive activities as well as facilitators for prevention during the process.

### Barriers to prevention

There were three main barriers: uncertainty about prevention, competing priorities and existing habits. Patients reported that, at first, they were uncertain about the value of preventive treatments:

"“Just getting used to some of the new techniques in the respect of, “We will not drill, we will do this and its okay”, that was a complete change from that point of view.”"

"“My biggest fear was that it was not going to work and it was going to be a waste of money.”"

Home care activities (tooth brushing and flossing) were seen as time consuming and not a priority:

"“I just get so busy with home and kids and stuff that it [tooth brushing and flossing] just comes down the ladder of priority a little bit.”"

Old habits were also hard to change:

"“Lifestyle changes are the most difficult, yeah, flossing everyday all the time especially. I think we are all guilty of it, we have routines and then we get sloppy sometimes and maybe miss things.”"

### Facilitators for prevention

Patients talked about important facilitators for prevention. These included having more treatment options, being able to go back to work without a numb lip after receiving dental treatment, gaining a new understanding about what they could do to take care of their teeth and being “treated as a person” by their dentist.

Patients were attracted to prevention because it gave them treatment options apart from restorative care:

"“The dentist has reassured me that I can strengthen the teeth that I have; so it was not just a matter of ripping out fillings and putting in new ones.”"

Patients also valued receiving dental treatment without consequent numbness from anaesthetic injections. By avoiding the “dead mouth feeling” after treatments, they could visit the dentist and go back to work afterwards, which was previously not possible. This made it easier to fit attendance into a patient’s schedule.

"“It is probably better in the way that you do not go away feeling sore or feeling numb with a dead mouth feeling and all that sort of thing. I used to take time off to come here where now I just make the appointment, work my day around it, jump in here, take off and just go straight to work.”"

Patients talked about gaining new knowledge and beginning to understand what they could do at home to manage their oral health.

"“They [dentist and dental team] helped me to understand a bit more that starting from before I go to the actual dentist I can start to take care of my teeth for a long time, even after I left the dentist from that appointment.”"

Once preventive knowledge was gained, it had to be put into practice. Some patients were not used to tooth brushing twice a day or flossing at least once a day.

"“I used to clean my teeth at night before I went to bed, sometimes in the morning, and I had to be more diligent than that, but I am probably still not diligent enough, but I try to clean them at least twice a day now.”"

They also had to visit the dentist more often for fluoride treatments and oral hygiene coaching.

"“I just think that if I have to go every 3 months or so to get fluoride put in to strengthen my teeth, I would rather do that then not go for 12 months and then I need a filling.”"

All these activities took time and were slowly incorporated into patients’ busy daily routines, which included taking care of their children, work and home duties. However, when patients perceived that dentists and members of dental team were genuinely listening to their concerns and making an effort to help them “keep their teeth” it made them feel respected and reassured, increasing their motivation to follow home care instructions and take responsibility for their teeth.

"“I think that I am treated in a more of a one person to person way, a bit more like the same level. It is not just assuming that I have the knowledge.”"

"“It is just more and more of a personal level than patient-dentist level and I feel more inclined to follow their instruction. Besides, I know now that if I do not look after my teeth I will be a lot worse off.”"

### What did patients value in their dental care?

All patients, regardless of the practice they came from or their level of clinical risk of developing dental caries wanted a caring dentist who would respect them and listen to their concerns without “blaming” them for their oral health status.

"“As in most things it is a two-way relationship. So it is the gentleness, it is the trust, it is the respect, it is actually the transparency that has being able to build up a relationship where you can trust your dentist to give you a very open and honest answer about any treatment.”"

"“I do not have knowledge but the dentist acknowledges that I am person of intelligence as well. So I suppose, it is how [the dentist] explains the information without making me feel like [the dentist] has been speaking to me condescendingly.”"

"“I have dropped dentists in the past. I think that how they were able to relate to me as a person was probably the biggest indicator of whether I felt comfortable with what they were doing. I suppose if you have a choice of five people with the same skill set, it is how they are able to deliver that skill set that is more important than the skill set as such.”"

When reflecting on their new preventive care experiences, patients suggested that there were two types of dentists and two different ways of practicing dentistry which we categorized as “old-school dentistry” and “new-school dentistry”. Patients described the “old-school” dentist as one who had a “mandate for doing fillings”, would not give patients preventive options and lacked communication skills. Some patients wondered if there was an “old-school institution” that graduated dentists without any knowledge of preventive options.

"“I wonder whether old-school dentists have got a mandate on what they do or whether that is easier or they make more money from continually filling teeth.”"

"“The dentists never mentioned to me any possibility of fluoride treatments. So I just think that there must be an old-school where this is the way it is done.”"

"“They [dentists] just think that you have got nothing else going on in your life and you are 100% focused on dealing with this one issue, which is just one facet of your life. They should listen to what patients say in the first place.”"

On the other hand, patients said they had also met “new-school dentists” over the years.

"“I have been fairly better educated in this practice. I used to just go to other dentist and get my teeth fixed and no one really ever said what to do in between.”"

"“I always think that it is better if the dentist explains it to you and shows you what to do. My dentist is quite proactive and supportive.”"

"“Dentists should at least offer the preventive treatment. Because I think there are a lot of people out there that do not have enough knowledge about the fluoride that you need. It is just too easy to say, “Okay that needs root canal” or “that needs to be removed” or, “that needs a filling” before it gets to that actual stage.”"

These “new-school dentists” were greatly valued. Patients valued “new-school dentists” because they educated patients, monitored and reassured them frequently during visits and made them aware of preventive options.

## Discussion

### Transferability of findings and limitations of the study

As with all qualitative research, judgments about the transferability of these findings to other settings rely on understanding the context of this study. This was a study of private dental practices in the state of NSW, Australia – where dental services are overwhelmingly delivered in the private sector and not integrated into the medical system [5]. Dental practices in this study appeared to be more or less typical of Australian private practices. It seems likely that these results will be readily transferable to other private general dental practices in Australia and jurisdictions where the characteristics of practices and funding systems are similar. The degree to which they are transferable to other clinical or political contexts is a question for future empirical investigation.

The patients in the study had private dental insurance; they were used to visiting the dentist once to twice a year for check-up appointments and for restorative treatment when needed. They were not used to being treated by a dental hygienist. These practice and patient characteristics are similar to the Australian average, based on the results of The National Survey of Adult Oral Health 2004–06 NSW [7].

As in all qualitative research, the patients in this study were selected because they were expected to be information-rich cases, rather than as being representative of a broader population. As previously discussed, the sample was made up of people who had been exposed to structured preventive care, with a wide range of oral health states from high risk to low risk of developing dental caries as assessed using a standard instrument during the RCT. Patients were all attending a dentist and participating in a structured preventive program; people who rarely or never attend the dentist may respond differently. As in most research, there may be some selection bias resulting from patients having to actively opt-in to the research process (that is, being willing to participate and replying to the invitation letter.).

### Brief overview of findings and its relevance to the dental literature

During this study we developed a better understanding of how patients experienced dental care. Historical, biological, financial, psychosocial and habitual dimensions of patients’ experience were revealed (Table 3 and 4). We saw marked differences between patients’ experience of dental care in dental practices with and without a structured approach to prevention in place. Patients transitioned from their initial state of being trapped in a situation of having degenerating teeth through a stage where they had achieved lifestyle changes and experienced reinforcing outcomes. Through this process patients gained new knowledge, developed new clinical relationships and established new practices. Patients were amazed by their experience of dental care without “drilling and filling” teeth and characterised dentists as either “old-school” or “new-school” based on the treatment options provided and the clinical relationship offered.

This study suggests that oral health self-care was not simply a matter of individual patients changing their behaviour. Despite the existing barriers for prevention, changes occurred in the context of a relationship with a dentist and the dental team – having a preventive structured approach in place helped individual patients to feel that their dentist respected their views and concerns. This is consistent with literature that suggests that patients’ perceptions of the quality of dental care and the likelihood of them seeking care are related to their perceptions of dentists as caregivers [15]. Several studies have described perceived characteristics of dentists that are likely to increase care-seeking or satisfaction with care, including communication skills, informing patients about treatment options, and dental teams’ behaviour during dental visits [13-17,22-25].

Patients have been shown to have confidence in dentists who are friendly, kind, not victim blaming towards patients, are patient focused rather than income focused; and who take time to explain procedures [15,16]. Similarly, in our study, patients talked about being compliant with preventive care recommendations because they felt they were being “treated as a person and not as a patient.” There was a perception that the offer of preventive care was a caring action: by making this offer, the dentist demonstrated that he or she was committed to work with a patient to “keep their teeth.” In contrast, many patients wondered why their previous care had been mainly restorative, and were concerned that they had not been offered the benefits of preventive care earlier in life.

Despite having different clinical outcomes, patients in this study talked in very similar ways about what they wanted from their dental care experiences. Their evaluation of the dental care experience was simple: either they were respected as a person or not, offered a chance to keep their teeth or not. The importance of developing a respectful health care relationship and its implications for patients’ ability to feel respected, to become supportive of health care activities and to take action towards improving their health were previously pointed out by other authors [12]. We also observed that even when patients were uncertain about the value of a recommended treatment, a perception that their dentist cared about their problems persuaded them towards compliance. This suggests that even the most “uncooperative” [22-24] patient may have the potential to be more cooperative in the context of such a relationship.

### Concluding remarks

When structured preventive care was introduced, patients perceived the difference. This was true of patients in both practices, and for patients at all levels of risk of developing dental caries, that is, with healthy and less healthy mouths. Without preventive care, the existing vulnerability caused by a history of poor oral health was progressing to worsening oral health. People were either unable to pay for care and living with pain, or were continuously paying for restorative work; although they were unhappy with this situation, they felt unable to address it. Patients were initially uncertain about the effectiveness of structured prevention, and about their ability to implement it given competing priorities and existing habits. However this changed once structured preventive care had been experienced. Patients reported a new sense of ownership of their oral health, and no longer felt trapped in a situation of having degenerating teeth. They were now prepared to invest in an active program of oral health care. They appreciated some of the more concrete aspects of the new regimen, such as greater treatment choice and treatment without anaesthesia. But their motivation was substantially increased by their growing understanding of their oral health and what they could do to improve it. An even more significant motivator was a perceived change in the dentist-patient relationship: patients felt better respected. A key question to consider in concluding, then, is the degree to which this new sense of respect was dependent on dentists offering structured prevention. Surely, a dentist who offered only restorative care could provide a respectful and thus valued relationship as well.

Contrary to this, we argue that the respect that dentists offered and patients valued was intrinsically bound up with the provision of structured preventive care. This was so much the case that patients contrasted “old-school” and “new-school” dentists, the former offering only restoration and the latter offering structured prevention. “New- school” or “preventive” dentists were perceived as caring, non-judgemental, transparent and communicative. They provided patients with the knowledge and skills they needed to understand, take charge of, and self-manage their oral health. They offered monitoring, evidence-based information and reassurance rather than taking the automatic route of “drilling and filling” teeth. The very provision of prevention was seen to be a respectful act. Structured prevention – which necessarily involved more communication, education and skill development in patients – instituted a fundamentally different type of relationship between dentists and patients.

While all dental care – in fact, all clinical care – should be provided in a respectful manner, we propose that structured preventive care will be understood by patients to institute a particular and highly-valued type of respect. It seems unlikely that the kind of respect described here can be replicated inside a traditional, restoratively-oriented clinical encounter. The experience of having a dedicated, supportive and caring dental team helped patients to take control of their own oral health. These dental teams produced profound changes in not just the oral health care routines of patients, but in the way patients thought about their own oral health and the role of dental professionals. We believe that the distinction patients made between “old” and “new-school” dentists warrants further investigation, as does the relationship between prevention and respectful care. We conclude that, based on the results of this study, not only patients but private practice dentists have much to gain by reorienting their services towards systematic prevention.

## Abbreviations

RCT, Randomized Controlled Trial; DMFT, number of decayed, missing and filled teeth; NSW, New South Wales.

## Funding

The authors received financial support for the research from the following funding agencies: University of Sydney Postgraduate Award 2009; The Oral Health Foundation, University of Sydney; Dental Board New South Wales; Australian Dental Research Foundation; National Health and Medical Research Council Project Grant 632715.

## Authors’ contributions

All authors have made substantial contributions to conception and design of this study. AS carried out data collection, analysis, and interpretation of data. SMC made substantial contribution during data collection, analysis and data interpretation. AS, SMC, RWE, and AB have been involved in drafting the manuscript and revising it critically for important intellectual content. All authors read and approved the final manuscript.

## Competing interests

The authors declared no conflicts of interest with respect to the authorship and/or publication of this article.

## Pre-publication history

The pre-publication history for this paper can be accessed here:

http://www.biomedcentral.com/1472-6963/12/177/prepub
